# Impacts of heat exposure on pregnant women, fetuses and newborns: a systematic review and meta-analysis

**DOI:** 10.21203/rs.3.rs-4713847/v1

**Published:** 2024-07-18

**Authors:** Darshnika Lakhoo, Nicholas Brink, Lebohang Radebe, Marlies Craig, Minh Pham, Marjan Haghighi, Amy Wise, Ijeoma Solarin, Stanley Luchters, Gloria Maimela, Matthew Chersich

## Abstract

Climate Change has wide-ranging and severe health impacts, especially for vulnerable groups. We systematically reviewed the literature (n=198 studies) on heat impacts on maternal, fetal, and neonatal health, conducted meta-analyses to quantify impacts, analysed periods of susceptibility, and graded certainty. Studies covered 66 countries and 23 outcomes. Our results showed increased odds of preterm birth of 1.04 (95%CI=1.03, 1.06) per 1°C increase in heat exposure and 1.26 (95%CI=1.08, 1.47) during heatwaves. Similar patterns were shown for stillbirths and congenital anomalies. Gestational diabetes mellitus odds increased by 28% (95%CI=1.05, 1.74) at higher exposures, whileodds of any obstetric complication increased by 25% (95%CI=1.09, 1.42) during heatwaves. Patterns in susceptibility windows vary by condition. The review demonstrated that escalating temperatures pose major threats to maternal and child health globally. Findings could inform research priorities and selection of heat-health indicators. Clearly more intensive action is needed to protect these vulnerable groups.

### Background

Climate change induced impacts on health are increasingly evident. Record year-on-year temperatures pose major threats to vulnerable populations such as pregnant women and newborns.[1,2] Rises in temperatures and increase in extreme weather events are pushing both natural and human systems towards critical and irreversible tipping points.[3]

Anatomical and physiological changes during pregnancy alter thermoregulatory responses.[4,5] Increased cardiac output and plasma volume, coupled with heat generated by fetal metabolism and additional fat deposits collectively heightens the vulnerability of pregnant women to heat exposure, especially in the later stages of pregnancy.[4,6,7] Increased vasodilation and sweating assist in dissipating heat and maintaining a thermal gradient for heat loss from the fetus, but these responses are dampened if the mother has hypertension, haemorrhage, sepsis, or other underlying medical conditions.[4,5]

Heat-related risks during pregnancy include preterm birth,[8] stillbirth,[8,9] congenital anomalies,[10] small for gestational age,[11] gestational diabetes mellitus,[6] premature rupture of membranes,[6] increased hypertensive disorders,[12] and cardiovascular events[13] during labour. These conditions not only affect pregnancy outcomes, but also have long-term implications for the mother and child.[14] However, knowledge gaps remain, particularly in the quantification of risk, the breadth of heat-sensitive outcomes, the critical periods for susceptibility, risk factors for heat vulnerability, heat thresholds, and biological pathways.[6]

We aim to systematically review the literature and conduct meta-analyses to quantify the impacts of heat exposure on maternal, fetal, and neonatal health, identify windows of susceptibility, and grade the certainty of evidence. Quantifying the current burden of heat-related health impacts allows researchers to project future health and economic burdens under different scenarios of a collapsing climate. Understanding patterns can assist in establishing surveillance systems, developing targeted public health interventions, evaluating adaptation effectiveness, and informing policy decisions that protect pregnant women from the escalating risks. Any additional risk for adverse maternal and child health outcomes from heat exposure will have major implications for public health and the socio-economic conditions of affected individuals and communities.

## Results

We identified 19 587 records for screening, of which 198 were included in this review (Fig. 1). These studies, spanning 66 countries, and 6 continents (Table 1), show a growing interest in the field, with 84.9% published since 2010 (Extended data Fig. 1). While the majority of studies focused on high-income countries (63.3%) and temperate climate zones (40.1%), evidence covers a range of climates and regions (Fig. 2). A summary of findings table, and the 198 study references are available in Supplementary table S1 and file S2.

Table 1
Number and percentage of studies by continent, climate zone, year of publication, population group and income level.

| | Categories | Number of studies | Percentage (%) |
|---|---|---|---|
| **Location of study (continent)[a]** | Africa | 11 | 5.5 |
| | **Asia** | **68** | **34.2** |
| | Australia | 12 | 6.0 |
| | Europe | 36 | 18.1 |
| | North America | 59 | 29.6 |
| | South America | 9 | 4.5 |
| | Multiple | 4 | 2.0 |
| **Climate zone[b]** | A - Tropical | 10 | 5.1 |
| | B - Dry | 19 | 9.6 |
| | **C - Temperate** | **79** | **40.1** |
| | D - Continental | 21 | 10.7 |
| | Multiple | 68 | 34.5 |
| **Year of publication** | Pre-1980 | 4 | 2.0 |
| | 1980–1999 | 9 | 4.5 |
| | 2000–2009 | 17 | 8.6 |
| | **2010–2019** | **96** | **48.5** |
| | 2020–2023 | 72 | 36.4 |
| **Population group[c]** | Maternal | 52 | 23.9 |
| | Fetal and perinatal | 51 | 23.4 |

[a] N sums to 199. One paper Bakhtisityarava (2022) performs analysis separately on two different continents

[b] N sums to 199. One paper Qu (2021) reports estimates for two different climate zones

[c] N sums to 218. 18 papers report two outcomes, and one paper reports three outcomes Khodadadi (2022)

[d] N sums to 196. One paper Bakhtisityarava (2022) included three locations (Brazil, Mexico and Chile) with two different income levels. Three papers, Dieckmann (1938), Jensen (2013) and Wells (2002) are conducted in many countries

| | Categories | Number of studies | Percentage (%) |
|---|---|---|---|
| | **Neonatal** | **115** | **52.8** |
| **Income level[d]** | Low income | 6 | 3.1 |
| | Lower middle-income | 17 | 8.7 |
| | Upper-middle income | 47 | 24.0 |
| | **High income** | **126** | **63.3** |

[a] N sums to 199. One paper Bakhtisityarava (2022) performs analysis separately on two different continents

[b] N sums to 199. One paper Qu (2021) reports estimates for two different climate zones

[c] N sums to 218. 18 papers report two outcomes, and one paper reports three outcomes Khodadadi (2022)

[d] N sums to 196. One paper Bakhtisityarava (2022) included three locations (Brazil, Mexico and Chile) with two different income levels. Three papers, Dieckmann (1938), Jensen (2013) and Wells (2002) are conducted in many countries

Summary of effects

In total, 271 direction of effect estimates (positive/negative/not-reported) and 221 effect estimates (risk ratios, odds ratios, hazards ratios) were extracted across 23 outcomes. The most studied outcomes were preterm birth (n = 84), accounting for approximately one third of the total, low birth weight (n = 51), hypertensive disorders in pregnancy (n = 28), congenital anomalies (n = 22), and stillbirths (n = 19). The direction of effect was predominantly harmful, with opposing evidence in some instances, especially low birth weight, hypertensive disorders in pregnancy, and congenital anomalies, where findings show more heterogeneity (Fig. 3).

From the 221 effect estimates, 56 estimates quantified the impact of a 1°C temperature increase, 35 addressed the effects of heat wave exposure, and 130 compared the health outcomes under conditions of high versus low heat exposure. Figure 4 shows a summary of all exposure-outcome effect estimates. Notably, all effect estimates lie to the right of null, indicating increased risk, with the majority ranging between 1 and 1.5, thereby demonstrating a consistent increase in risk across varied exposure-outcome metrics.

Maternal outcomes

Heat exposure in pregnancy was associated with several adverse maternal outcomes. Hypertensive disorders, such as pre-eclampsia and gestational hypertension, were linked with heat exposure in 21 of the 28 studies. Notably, a study conducted in a Chinese cohort of over 2 million pregnancies showed consistent and significantly higher odds of hypertensive disorders with increasing heat exposure in the first and early second trimester, with a clear dose-response effect, OR = 1.16 (95%CI = 1.10, 1.22).[15] Six studies found significant heat impacts on increased hypertensive disorder risk in the first 20 weeks, with studies predominantly investigating the month of conception,[15–20] while three studies with a negative association measured heat exposure after 20 weeks, or in the month of childbirth and found a decrease in blood pressure with higher temperatures.[21–23]

All nine studies on gestational diabetes mellitus found a harmful association with heat exposure. A large study from Canada (n = 555 911) found that 10°C increase in mean temperature in the past 30 days was associated with 6% increased odds of gestational diabetes mellitus (95% CI = 1.05, 1.07).[24] In another large study in Taiwan (n = 371131) even higher odds per 1°C increase in heat exposure was reported (OR = 1.54 (95%CI = 1.48, 1.60)) when temperatures exceeded a 28°C threshold compared to lower temperatures.[25] Five of these studies found associations between gestational diabetes and heat in the second trimester, around the time screening typically occurs.[24,26–29]

Heat exposure was associated with increased risk of all-cause antenatal bleeding (n = 3), and placental abruption (n = 2), with short-term effects particularly evident.[30–32] Rammah et al. (2019)[31] found a 16% increased odds of placental abruption per 1°C increase in apparent temperature at a 1-day lag (95%CI = 1.03, 1.40). An additional maternal outcome linked to heat exposure was a 63% increased odds of prelabour rupture of membranes at higher heat exposure (95%CI = 1.23, 2.16) (n = 5). Infections (n = 4), specifically group B streptococci colonisation[33] and bacteriuria,[34] illustrated a harmful association with heat.

Mental health was assessed (n = 2) by screening for emotional stress, where a U-shaped association with temperature was demonstrated,[35] and secondly, by admissions for any mental illness, where a positive, but non-significant association was found.[36]

Exposure to heat, and especially heatwaves, was associated with increased all-cause emergency visits and hospital admissions across all five studies examined. Two studies found evidence of a dose-response effect; more extreme and longer-lasting heatwaves were associated with increasing odds of emergency visits and admissions.[36,37] Lastly, a positive, but non-significant association was found between heat exposure and delivery by caesarean section (n = 1),[38] and between increasing temperatures and increased risk of cardiovascular events, including stroke and cardiac arrest (n = 1).[39]

Fetal and perinatal outcomes

Heat exposure during pregnancy is associated with adverse fetal and perinatal outcomes, particularly stillbirths (n = 19), where harmful associations were found in over 10 million stillbirths. A meta-analysis of five USA based studies indicated a 14% increase in the odds of stillbirth for every 1°C rise (95%CI = 0.99, 1.32; $I^2$ = 93%) (Extended data Fig. 2a). This was similar to the 13% increase in studies comparing high versus low heat exposure (95%CI = 0.95, 1.34; I2 = 83%) (Extended data Fig. 2b).

Congenital anomalies demonstrated a notable but more heterogeneous association with high temperatures, with five of the 22 effect estimates demonstrating a protective association with heat. The study with the largest sample size of over 2 million women and 29 000 anomalies conducted in the USA, found a dose effect response with extreme heat (above 95th percentile) associated with increased odds of total anomalies by 29% (95%CI = 1.21, 1.38).[40] Most studies detected impacts of heat with exposure during the first few weeks of pregnancy,[41–43] while one study found no association when temperature was examined in the whole first trimester.[44] Meta-analysis assessing the impact of high versus low heat exposure on the odds of any congenital anomaly found an increased OR = 1.48 (95%CI = 1.16, 1.88, $I^2$ = 17%) across six studies (Extended data Fig. 3).

For spontaneous abortion (n = 7), despite most studies indicating a harmful direction of effect, the evidence lacks consistency, with five studies yielding no statistically significant association for spontaneous abortion,[36,45–48] and one study exhibiting an inverse U-shaped association.[49]

Additional findings include significant impacts of heat on conditions such as oligohydramnios, observed in two studies with relatively small sample sizes but notable for their agreement on a short (up to four days) lagged effect.[50,51] Non-reassuring fetal status, encompassing outcomes like fetal hypoxia and fetal growth restriction, consistently presented harmful associations with heat (n = 5). In the Gambia, for example, increased fetal strain (heart rate greater than 160 beats per minute and increased umbilical artery resistance) was associated with a 12% increased odds for each 1°C increase in heat exposure (95%CI = 1.03, 1.21).[52] Perinatal mortality risk increased by 53% with exposure to temperature above the 95th percentile in Spain RR = 1.53 (95%CI = 1.16, 2.02),[53] while a study on the same topic in Sweden utilising data from 1800’s, had similar findings, though non-significant.[54] Lastly, one study found a reduced placental weight and volume with high temperature exposure in the third trimester.[55]

Neonatal outcomes

Neonatal outcomes are affected by heat exposure, especially preterm birth, which is the most extensively studied condition, with 78 of the 84 effect estimates reporting a harmful direction of effect. A study with the largest sample size, analysing 56 million USA births, identified a reduction in gestation with exposure to extreme heat days, with an estimated annual loss of 150 000 gestation days and 25 000 infants born earlier.[56] Overall, a compelling body of evidence across diverse geographical locations and populations established a dose-response relationship, where risks for preterm birth rise in tandem with increases in heat exposure.[55,57–61]

In the meta-analysis (Fig. 5), the odds of a preterm birth rose by 4% (95%CI = 1.03, 1.06; $I^2$ = 85%) per 1°C degree increase in temperature across all the study locations with an exposure lag of less than four weeks (short lag). While there is substantial heterogeneity across these studies, all the effect estimates have the same direction of effect. Similarly, with heatwave exposure, the odds of preterm birth increased by 26% in meta-analysis (95%CI = 1.08, 1.47; $I^2$ = 63%) (Fig. 5). When comparing high versus low heat exposure, the odds of preterm birth across all studies was 12% at higher heat exposure with a short lag (95%CI = 1.06, 1.18; $I^2$ = 92%) (Extended data Fig. 4). There is significant heterogeneity in this meta-analysis, with two studies that have a protective direction of effect.

When exploring windows of susceptibility, we see varying evidence across the literature, possibly suggesting more than one vulnerable exposure window may be present for risk of preterm birth. Studies of heat exposure with a longer lag period (an exposure to high temperatures more than four weeks prior) were larger than with short exposure.[62-66] Specifically, in the meta-analysis, the odds of preterm birth were 37% higher (95%CI=1.08, 1.74; $I^2$=98%) at high heat exposure, across all studies for a long lag (Extended data figure 5).

Subgroup analysis showed that the impacts of heat exposure on preterm birth risk vary by country income level, with the highest risk in low-income countries OR = 1.61 (95%CI = 1.39, 1.86) compared to upper-middle income OR = 1.10 (95%CI = 1.00, 1.21) and high-income countries OR = 1.11 (1.06, 1.15) (Fig. 5) at high versus low heat exposures.

In addition to studies presenting odds or risk, several studies have quantified the number of preterm births associated with heat, attributed preterm births to climate change, and quantified the economic burden. In Australia, an excess of 11 (95%CI = 9, 13) per 10,000 liveborn are preterm due to immediate heat stress and 36 (95%CI = 29, 43) per 10,000 excess preterm births are due to cumulative (lag0 to 6 days) heat stress. [67] One attribution study and economic evaluation in China found that heatwave related preterm births induced by anthropogenic climate change, resulted in an average of 4609 (95%CI = 711, 6110) cases annually. [68] The total economic costs of human capital losses caused by anthropogenic climate change on preterm birth are expected to exceed $1 billion annually in China. [68]

Heat exposure was associated with increased odds of low birth weight, however, there was heterogeneity in this outcome, with six of the 51 effect estimates having a protective effect. The largest study, based on 34.7 million births in the USA,[69] found that every additional day with mean temperature between 26.7–32.2°C in the preceding nine months, increased very low birth weight by 0.008 per 1000 (0.1% of mean, $p < 0.5$), particularly among Black and Hispanic mothers. Meta-analysis found the odds of low birth weight 29% higher (95%CI = 1.04, 1.59; $I^2$ = 95%) at higher versus lower heat exposure across 12 studies (Extended data Fig. 6).

Additional neonatal outcomes assessed were small-for-gestational-age (n = 6), neonatal admissions (n = 4), neonatal morbidity (n = 6) (jaundice, respiratory distress syndrome, low Apgar scores), and neonatal mortality (n = 1), which were all adversely associated with heat. Neonatal admissions were at higher odds with exposure to heatwaves; from 33% (95%CI = 1.27 to 1.38) in Brazil to 43% (95%CI = 9.2, 88%) in India.[37,70]

Composite outcomes

We derived five composite outcome groups from the data:

1. Pregnancy specific medical disorders – gestational diabetes mellitus, hypertension in pregnancy and cardiovascular disease related to pregnancy
2. Obstetric complications – antenatal bleeding, preterm birth, prelabour rupture of membranes
3. Pregnancy loss – spontaneous abortion, stillbirths
4. Fetal growth effects - intrauterine growth restriction, small-for-gestational age, low birth weight
5. Healthcare system burden - caesarian section, maternal and neonatal admissions

In meta-analyses, the average odds of obstetric complications increased by 5% (95%CI = 1.03, 1.06; $I^2$ = 92%) for every 1°C increase in temperature, and by 25% (95%CI = 1.09, 1.42; $I^2$ = 59%) with exposure to heatwave (Extended data Fig. 7). The other composite outcomes were not suitable for meta-analyses due to the extent of heterogeneity.

Certainty grading

Using an adapted approach to the International Panel on Climate Change confidence assessment, the evidence scores of type, quantity, quality, and consistency across reported outcomes are shown in Extended data Table 1.

For quantity, the outcomes that have the most evidence are for preterm birth, low birth weight, hypertension in pregnancy, congenital anomalies and stillbirths. Most studies have consistent results, apart from the body of evidence on congenital anomalies and hypertension in pregnancy that score poorly. Accounting for levels of agreement, and evidence, the outcome with a very high certainty was preterm birth. Outcomes with high certainty were gestational diabetes mellitus, hypertension in pregnancy, stillbirths, and neonatal admissions. Outcomes with limited evidence and low agreement, that had very low confidence were caesarean section, placental outcome, and neonatal mortality.

## Discussion

This systematic review collated evidence from across the world on the harmful impacts of heat on maternal, fetal, and neonatal health, significantly advancing our understanding of the vulnerabilities associated with heat exposure during pregnancy. Five critical outcomes - preterm births, low birth weight, hypertension in pregnancy, congenital anomalies, and stillbirths accounted for 75% of the evidence. Ten outcomes, however, including several major causes of maternal mortality, were represented by only one or two publications, constraining our ability to summarise the full extent of heat harms in these populations.

Maternal outcomes only account for a quarter of the body of literature in this review and are under-represented in heat-health research in general. Evidence is, however, robust for increased risk of hypertensive disorders in pregnancy (high confidence) and gestational diabetes mellitus (high confidence). Though few studies examined infectious disease outcomes, a large body of work outside of maternal and neonatal health suggests that heat-related exacerbations of infectious diseases may pose a profound threat.[1]

Most studies on heat-related fetal outcomes had addressed risk of stillbirth (high confidence) and congenital anomalies (medium confidence). Similar increased risk of stillbirth is reported in Chersich et al. (2020),[8] with the inclusion of an additional 11 studies in this updated review. Preliminary findings also suggest potential heat impacts on non-reassuring fetal status (medium confidence) and spontaneous abortions (low confidence), which broadens the scope of enquiry into heat impacts on fetal health and provides insights into the underlying causal pathways that mediate adverse outcomes.

By far, the most evidence is available for the adverse impacts on neonatal outcomes, particularly preterm birth (very high confidence) and low birth weight (medium confidence), both crucial determinants of child mortality, health, and wellbeing over the life course. The evidence suggests a dose-response relationship for preterm birth. Importantly, there have been early efforts to quantify attribution to climate change and its consequent economic burden, [68] with important implications for loss and damage funding, advocacy and monitoring. Our review also provides some evidence for heat impacts in understudied outcomes such as neonatal morbidity (medium confidence) and neonatal admissions (high confidence).

While windows of vulnerability during pregnancy were identified for some outcomes, much remains unknown. For preterm birth, two periods of vulnerability were identified, whereas for hypertension in pregnancy, the very early gestational period was identified as a critical period, likely related to the early placentation period. Very short-term lags were identified for hospital admission, antenatal bleeding, and prelabour rupture of membranes, likely linked to their pathophysiological pathways. Gestational diabetes was strongly linked with heat during the second trimester. This may reflect the timing of screening for diabetes in pregnancy, rather than a heightened period of vulnerability. This is one example of potential bias with opportunistic reanalyses of existing data. More robust information on vulnerability windows in pregnancy and lag effects would help target protective interventions, especially heat-health messaging for pregnant women.

To explain the harmful impacts of heat during pregnancy, various pathophysiological mechanisms have been proposed, mostly based on animal studies.[9] Putative mechanisms include elevated maternal body temperatures (including from infection related fever), maternal dehydration leading to electrolyte imbalances, endocrine system dysregulation, altered glucose metabolism, and release of anti-diuretic hormone, oxytocin, adrenaline, and stress hormones.[52,71-73] The upregulation of heat shock proteins may also play a role in promoting cytokine release and tissue inflammation contributing to premature onset of labour and placental insufficiency.[74,75] A series of studies, funded by the Wellcome Trust, is underway to understand the biological mechanisms of heat vulnerability in pregnancy https://wellcome.org/grant-funding/schemes/biological-vulnerability-extreme-heat-maternal-and-child-health, https://www.high-horizons.eu/.

The growing body of evidence allowed an initial exploration of composite outcomes, which are useful from a public health perspective as they may capture the overall burden of disease and allow for the quantification of impact on related disease processes (e.g., conditions related to dehydration or fluid shifts), and in specific periods of pregnancy or childbirth. Understanding where the principal burdens lie will help inform decisions around whether to focus on home-based interventions in late pregnancy, or on facility cooling, for example.

Our review identified significant research gaps, particularly in tropical climate zones, and the Global South more broadly. This lack of evidence, from the areas most affected, makes it challenging to mobilise adequate resourcing, or to target resources effectively. Data gaps may reflect differentials in research resourcing and capacity, but also in data quality and the availability of electronic health record data for reanalysis. Novel approaches such as systematically collating individual-level participant data from cohorts and trials,[76] and specific initiatives to extract data from paper-based records, may be the only means of adequately addressing these gaps in the short term. These efforts, in turn, support the longer-term goal of establishing formal indicator systems that track burdens and progress with adaptation responses.

The systematic review has several limitations. The updated search was limited to PubMed and citation searching. Though the large majority of papers had been found in the initial search through this approach, some studies may still have been missed. Additionally, we did not conduct a formal risk of bias assessment of included studies, given that the original review noted predominately high risk of bias ratings. We elected to rather use an IPCC tool to grade certainty.

Our systematic review is limited by considerable heterogeneity in heat exposure metrics, outcome measures, and study designs, which complicated data synthesis and interpretation. Meta-analyses were limited to studies that provided directly comparable effect estimates. Further, many meta-analyses had high $I^2$ values which require cautious interpretation. Employing multiple synthesis methods in this review aimed to triangulate results to minimize biases inherent in each approach. Standardised methodologies and reporting guidelines in this field may enhance comparability and validity of findings.

In summary, this systematic review demonstrates clear and sizable linkages between heat exposure and multiple adverse maternal, fetal, and neonatal health outcomes. The gaps in evidence on key outcomes and specific locations, however, and the lack of long-run systematic measurement of these harms signal a failure to plan and protect pregnant women, newborns, and other vulnerable populations against climate change. Interventions to be prioritised now may include issuing timeous early warnings, intensive cooling and hydration initiatives during heat waves, behaviour change awareness, cooling in health facilities, and enhanced surveillance. More broadly, it will require the health sector and other sector leaders to exert real influence over health, urban planning, housing, transport and the energy sector to reduce heat exposure, to avert further climate collapse and to fulfil their duty of care to pregnant women and the next generation.

## Online methods

### Study design

We conducted a systematic review following the 2020 Preferred Reporting Items for Systematic Reviews and Meta-Analyses (PRISMA) guidelines.[77] Included were all peer-reviewed epidemiological studies assessing the impact of heat during pregnancy on maternal and neonatal outcomes. This review forms part of a larger systematic mapping exercise that described the body of heat-health impacts and adaptation intervention literature.[78] We also include data from systematic reviews, conducted as part of the systematic mapping by Chersich et al. (2020),[8] and Haghighi et al. (2021).[10] The reviews are registered in PROSPERO under CRD42019140136, CRD42018118113, and CRD42020173519.

### Search strategy and data extraction

We utilised a search strategy that was developed in MEDLINE (PubMed) and adapted it for Web of Science, Science Citation Index Expanded, Social Sciences Citation Index, and Arts and Humanities Citations Index. The systematic mapping search was conducted in September 2018 and updated in MEDLINE in 2019 and July 2023. The full search strategy can be found in Chersich et al. (2016)[79] and supplemental file S3. We included all study designs apart from systematic reviews and qualitative studies. No date restrictions were applied, and no grey literature was included. The original review in 2018 involved independent, duplicate screening of titles and abstracts, and data extraction from full text articles. For the updated review, screening and extraction was done by a single reviewer. EPPI-Reviewer software was used for reference management, screening and data extraction.[80] We extracted study citation identifiers, location, sample size, main study outcome and effect estimates. The study location was used to establish country-level gross domestic product (https://databank.worldbank.org/source/world-development-indicators) and Koppen-Geiger climate zones.[81]

### Synthesis

We utilised multiple synthesis methods to accommodate for heterogeneity, using SWiM Guidelines[82] and the Cochrane Handbook.[83] First, we grouped all the studies thematically by outcome type and population group, as per the WHO conceptual framework on extreme heat on maternal, newborn, and child health (https://www.high-horizons.eu/conceptual-framework-on-extreme-heat-and-maternal-newborn-and-child-health/), utilising Global Alignment of Immunization safety Assessment in pregnancy (GAIA) preferred terminology.[84] Vote counting illustrated the number of direction of effect measures, demonstrating benefit or harm per outcome group. Meta-analysis grouped studies based on heat exposure metrics and exposure lags, and were presented where clinically, and statistically appropriate. Odds ratios (OR), hazard ratios and risk ratios were considered comparable, as per Cochrane handbook. We selected three heat exposure metric groups as per Chersich et al. (2020),[8] 1) odds of outcome per 1°C increase in temperature, 2) odds of outcome during heatwave versus non-heatwave period, and 3) odds of outcome at high versus low heat exposure. Effect estimates were transformed where appropriate to meet these three grouping, for example Fahrenheit converted to Celsius and per 10°C calculated to per 1°C increase. We further grouped preterm birth and stillbirths based on lag, where short-term lags account for exposure during the four weeks preceding the event, and long-term lags beyond that time. We calculated the standard error (SE) of the effect estimate using the upper and lower confidence intervals (CI). Where the upper and lower bound SEs differed, we averaged the two and generated a new CI based on the estimated SE. Heterogeneity was explored through subgroup analyses where feasible. We evaluated the meta-analyses by interpreting a combination of the $I^2$ value, the difference between common and random effects models, funnel plots, and prediction intervals for outcomes with at least nine measures. Where meta-analysis was deemed inappropriate, the median OR and interquartile range was presented instead. All descriptive and meta-analyses were conducted using R version 4.3.1, with the meta package 6.5-0.[85]

### Certainty grading

We adapted the IPCC system of calibrated uncertainty language , to evaluate evidence type, quantity, quality, consistency (ranked limited, medium or robust), and degree of agreement (low, medium or high) to express levels of confidence (from very low to very high) for each outcome.[86] We chose the IPCC grading system over GRADE, and other similar tools due to its suitability for climate-related research. This approach is suitable for observational data, which might be undervalued by systems like GRADE that often rate such studies as low quality. For type, all observational data was ranked medium, above expert opinions or case studies and below randomised controlled trials. Quantity was assessed by the number of studies per outcome. Previous reviews have showed that nearly all observational studies are at high risk of bias, therefore we did not assess quality or risk of bias in this study.[8,14,87] Consistency was measured by calculating the ratio of harmful to protective results. Agreement was determined by surveying four study authors and reviewers to apply their expert knowledge in the field to categorise each outcome as low, medium, or high. Confidence statements were allocated based on evidence and agreement scores. The final agreement scores and confidence statements were reviewed and confirmed by consensus by DPL and MFC.

### Declarations

## Author contributions

**Darshnika Lakhoo**: Conceptualisation, methodology, validation, formal analysis, investigation, data curation, writing – original draft, writing – review and editing, visualisation, project administration. **Nicholas Brink**: Methodology, software, validation, formal analysis, investigation, data curation, writing – original draft, writing – review and editing, visualisation. **Lebohang Radebe**:

Methodology, software, validation, formal analysis, investigation, data curation, writing – original draft, writing – review and editing, visualisation. **Marlies H Craig**: Methodology, formal analysis, investigation, data curation, writing – original draft, writing – review and editing, visualisation. **Min Duc Pham**: Methodology, validation, investigation, writing – review and editing. **Marjan M Haghighi**: Methodology, validation, formal analysis, investigation, writing – review and editing. **Amy Wise**: Methodology, investigation, writing – original draft, writing – review and editing. **Ijeoma Solarin**: Methodology, validation, investigation, writing – review and editing. **Stanley Luchters**: Investigation, writing – review and editing. **Gloria Maimela**: Investigation, writing – review and editing. **Matthew F Chersich**: Conceptualisation, methodology, validation, formal analysis, investigation, writing – original draft, writing – review and editing, visualisation, supervision, funding acquisition. **Heat-Health Study Group**: Validation, investigation, writing – review and editing.

## Conflicts of interest

DPL, NB, MC and MFC hold investments in the fossil-fuel industry through their pension funds.

## Acknowledgements

We would like to acknowledge the contributions of Debra Jackson, Birgit Kerstens, and Nathalie Roos, who reviewed this work as part of a project deliverable in HIGH Horizons. Similarly, we would like to acknowledge Veronique Fillipi who contributed to the literature search and data extractions.

This project has received funding from the European Union's Horizon Framework Programme under Grant Agreement No. 101057843. Project partner LSHTM is funded by UKRI Innovate UK reference number 10038478. Further, this work is supported by the Fogarty International Center and the National Institute of Environmental Health Sciences (NIEHS) and OD/Office of Strategic Coordination (OSC) of the National Institutes of Health under Award Number U54TW012083. Lastly, this work was supported by the Wellcome Trust [227204/Z/23/Z]. The content is solely the responsibility of the authors and does not necessarily represent the official views of the European Commission, UKRI, National Institutes of Health and Wellcome Trust.

## Data availability statement

The data supporting the findings of this study are available within the paper and its supplementary information files. The analytical code is available upon request to corresponding author.

Supplementary information is available for this paper.

Correspondence should be addressed to Darshnika Lakhoo at Darshnika.Lakhoo@witsphr.org

## Figures

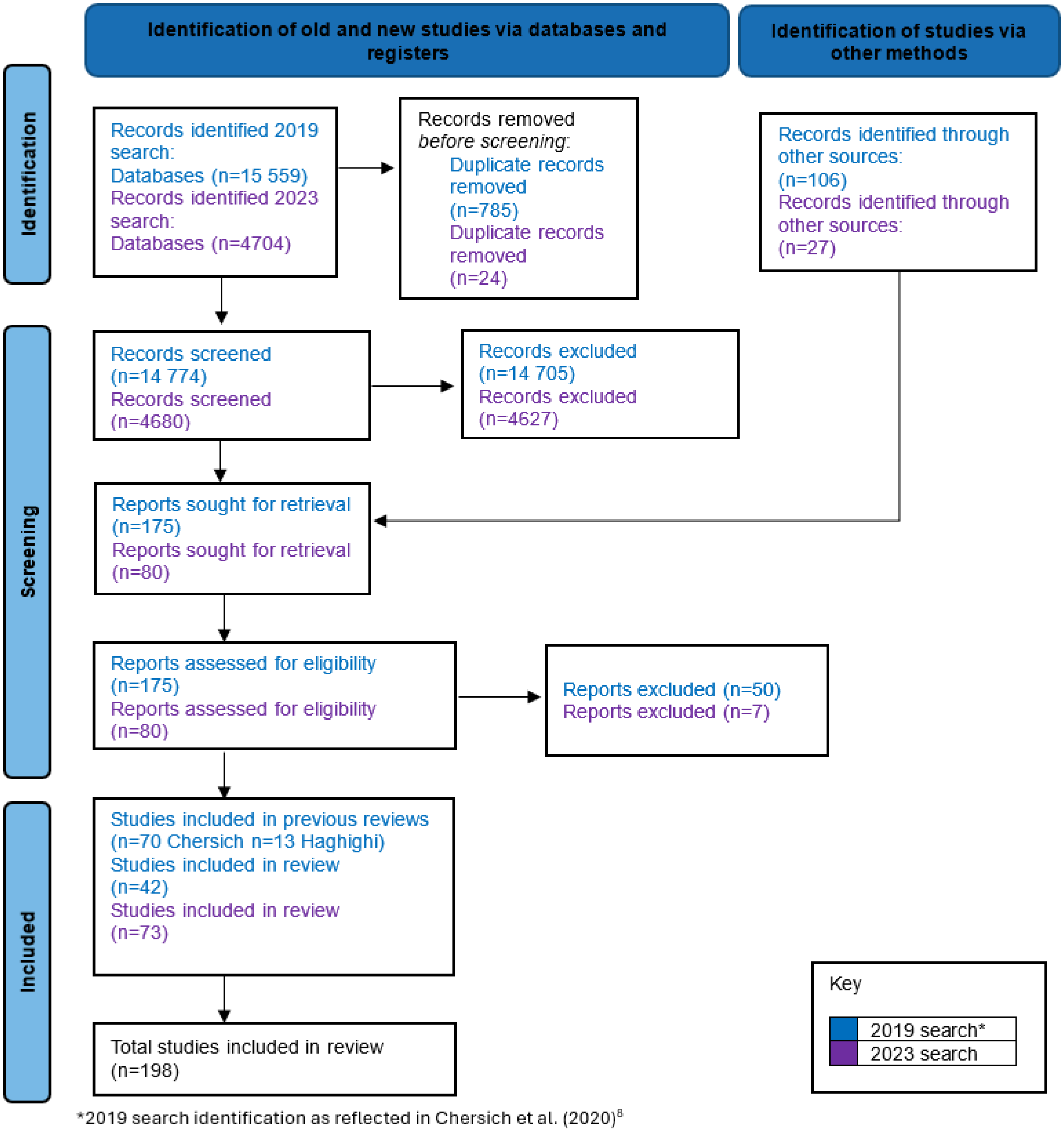

### Figure 1

Adapted PRISMA diagram representing study selection process from 2019 and 2023 searches

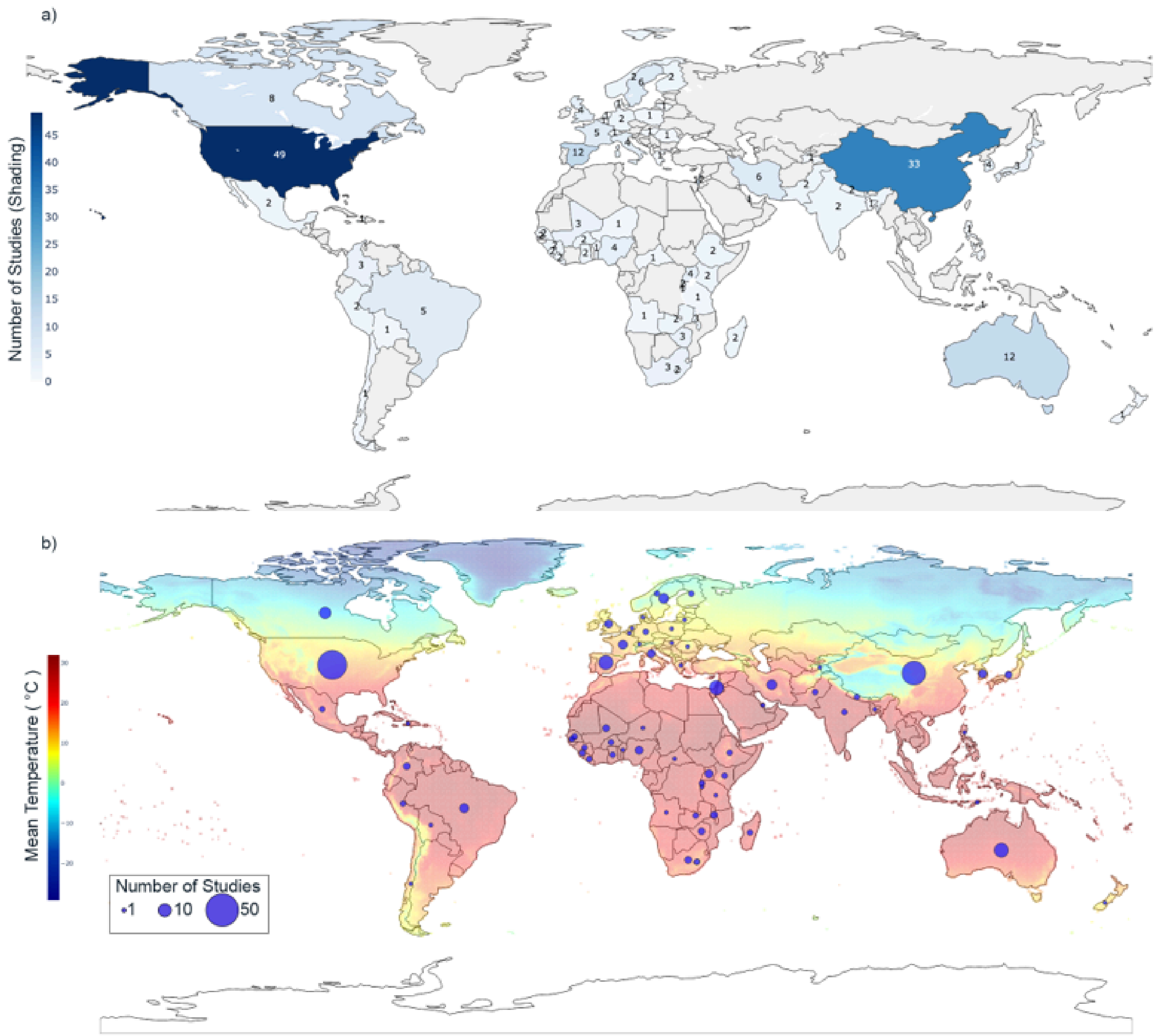

Figure 2

Distribution of studies by a) country and b) annual mean temperature

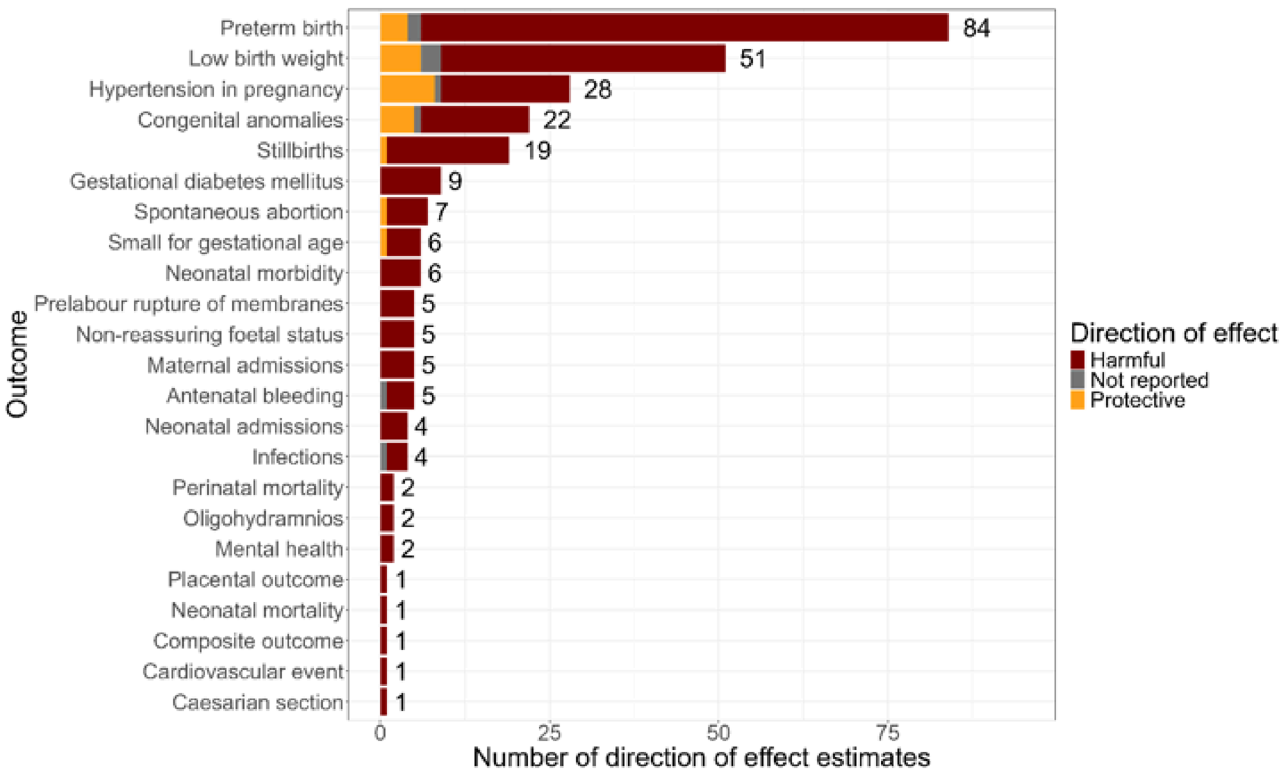

**Figure 3**

**The 271 direction of effect estimates as a result of heat exposure in pregnancy, per outcome category (vote counting)**

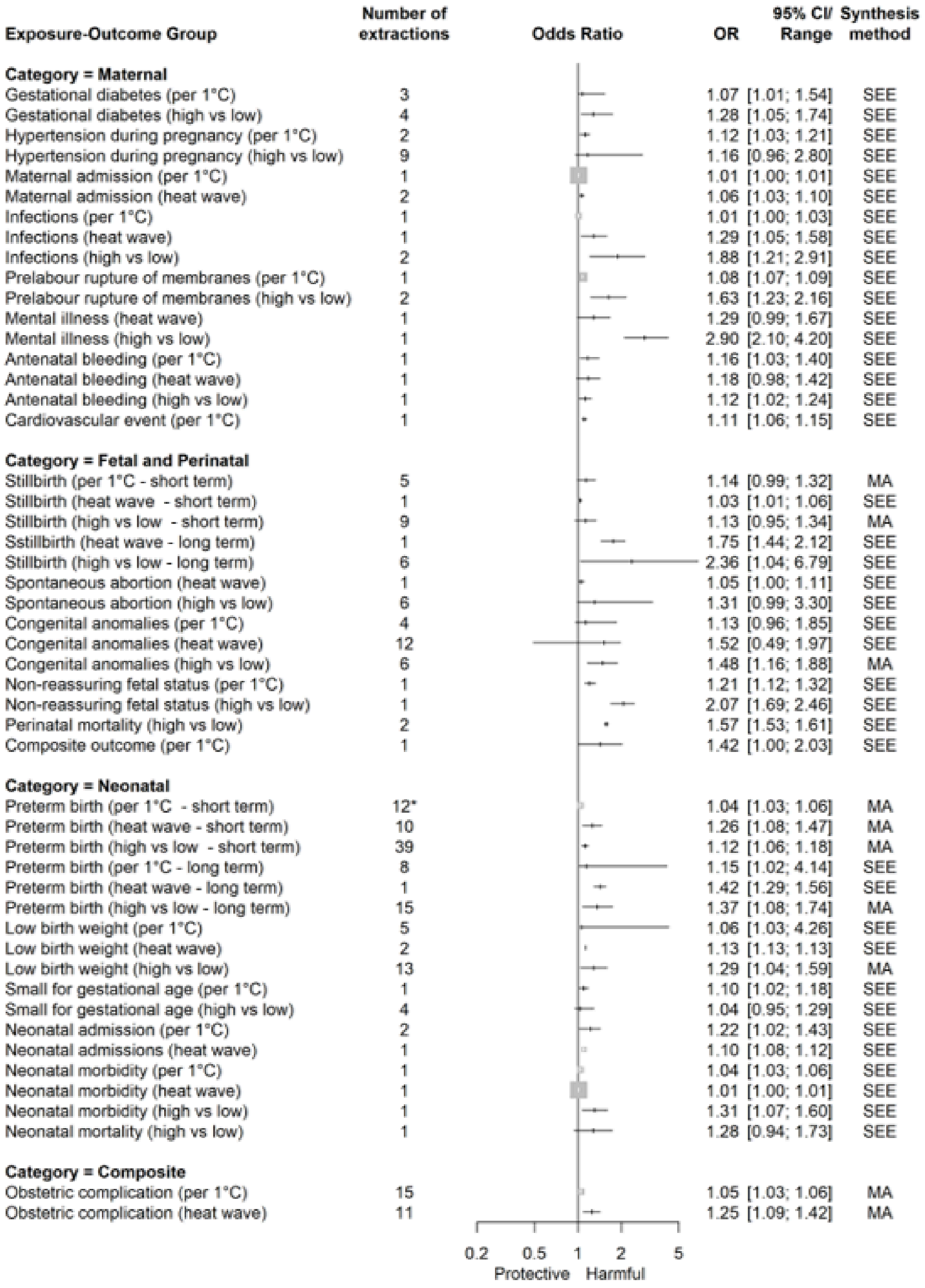

*MA – meta-analysis, SEE- summary of effect estimates*

### Figure 4

Forest plot representing a summary of effect estimates for each exposure-outcome group.

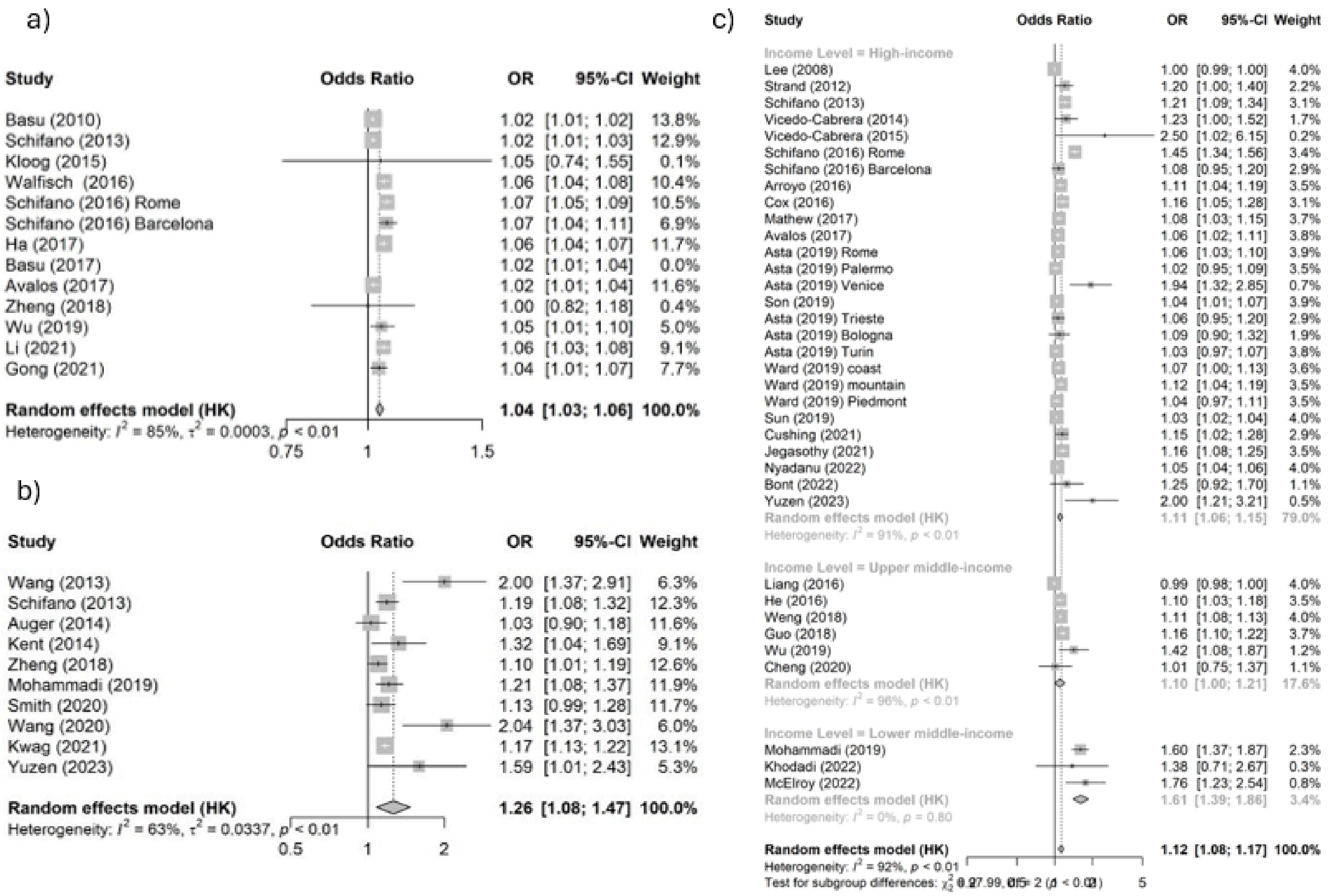

#### Figure 5

**Meta-analysis of odds of preterm birth with heat exposure in the preceding four weeks a) Per 1°C increase in temperature b) Heatwave c) Sub-group meta-analysis at high versus low heat exposure, split by country income level**

## Supplementary Files

This is a list of supplementary files associated with this preprint. Click to download.

- Supplementalfilesforsystematicreviewandmetaanalysismanuscriptfinal.docx
